# An Approach to Self-Assembling Swarm Robots Using Multitree Genetic Programming

**DOI:** 10.1155/2013/593848

**Published:** 2013-06-18

**Authors:** Jong-Hyun Lee, Chang Wook Ahn, Jinung An

**Affiliations:** ^1^Department of Computer Engineering, Sungkyunkwan University (SKKU), Suwon 440-746, Republic of Korea; ^2^Robot Research Division, Daegu Gyeongbuk Institute of Science & Technology (DGIST), Daegu 711-873, Republic of Korea

## Abstract

In recent days, self-assembling swarm robots have been studied by a number of researchers due to their advantages such as high efficiency, stability, and scalability. However, there are still critical issues in applying them to practical problems in the real world. The main objective of this study is to develop a novel self-assembling swarm robot algorithm that overcomes the limitations of existing approaches. To this end, multitree genetic programming is newly designed to efficiently discover a set of patterns necessary to carry out the mission of the self-assembling swarm robots. The obtained patterns are then incorporated into their corresponding robot modules. The computational experiments prove the effectiveness of the proposed approach.

## 1. Introduction

When robots try to successfully complete their mission in various environments, it is necessary to retain high autonomy and intelligence like humans. Robots should employ precise sensors and complex controllers and mount high performance processors in order to attain complete autonomy and intelligence. However, these enhanced devices bring forth the extremely expensive cost in constructing an autonomous robot system. Furthermore, the efficiency of the autonomous robot system dramatically decreases as the working space enlarges [1].

There is a well-known approach to solving the previously mentioned problems, which utilizes numerous robots by adopting swarm intelligence. Swarm intelligence makes a swarm of robots perform their tasks in collaboration with themselves. It denotes that the swarm robots can have a lot of advantages: stability, scalability, robustness, efficiency, and so on. Swarm robotics is a field of research on the swarm of robots, which is working in conjunction with the nature-inspired algorithms (i.e., swarm intelligence). The swarm robotics aims to develop an all-round autonomous machine in various practical areas such as industry, agriculture, fishery, military, and medical. In recent years, many studies have been carried out on the construction, exploration, national defense, and security fields. In fact, most of the real-world autonomous problems include complex situations and broad working space. Thus, the swarm robotics can be applied to a lot of areas in the sense of improving efficiency on the cost of installation and maintenance [2].

Nevertheless, the swarm robotics still has some problems when applying to practical applications due to the hurdles of the current technologies in constructing the promising self-assembling swarm robots (i.e., module robots). For instance, the motors of module robots lack physical strength. The battery which is contained in the modules cannot be as small as a microsize. A lot of researchers have tried to improve the module robots in order to find a better mechanism for the system [3–5]. In this sense, this paper develops a new control mechanism for the swarm robots by using evolutionary techniques (i.e., genetic programming) as an effort to get over these limitations.

The rest of this paper is organized as follows. In Section 2, we introduce the fundamental knowledge on the self-assembling swarm robots, the oscillator that is the core controller of the system, and the genetic programming (GP). In Section 3, we present the proposed evolutionary self-assembling mechanism for the swarm robots. The experimental results are shown in Section 4. Finally, we conclude this paper in Section 5.

## 2. Related Work

### 2.1. Self-Assembling Swarm Robots

 A self-assembling swarm robot system (*S*) consists of robot modules (*M*s), which are a kind of robotic cell, denoted by *S* = {*M*_1_, *M*_2_,…, *M*_*N*_}, where *N* is the number of module robots. The self-assembling in the swarm robotics means that these module robots are able to be combined together in an efficient manner. Each module has two major types of equipments: *motion* and *connection* devices. The former such as rotation motor and joint devices offers the motion power and decides the degree of freedom (DOF) of the system. The latter such as magnet, ring, and hook devices connects the modules together. Also, each module robot requires the inclusion of auxiliary components such as battery, controller, processor, and sensors, which are necessary for the distributed control [6–9]. In principle, the system can transform its own shape into another one suitable to carry out given tasks. Therefore, it can conduct the work which cannot be carried out by a single module because all the robot modules operate in cooperation with themselves. For example, the system may be transformed into the shape of a snake to pass through a pipe. It is also possible to convert the system into the shape of a four-legged animal to walk on rugged roads or climb stairs.

### 2.2. Oscillator

 A central pattern generator (CPG) makes the oscillators define their signal patterns. This CPG concept came from the biological understanding of the neural network in human's brain. In other words, the robots system is to a human and the CPG is to a brain. The CPG is a key element to determine the performance of a system. Thus, the main target of this study is to develop a new mechanism that plays a role of CPG by means of genetic programming (GP).

The oscillator contained in each module is the main issue in the self-assembling robot system that is to be controlled in a distributed fashion [5, 6]. This can be inspired by the observation that each gait of animals works at regular intervals to walk. In this sense, a lot of locomotive robots in recent days are based on the idea of using oscillators, and thus most of recent self-assembling robots have employed oscillators [10–15]. In general locomotive robots, each joint of the robots has its own oscillator which generates a signal for the motion of that joint.

**Figure 1:**
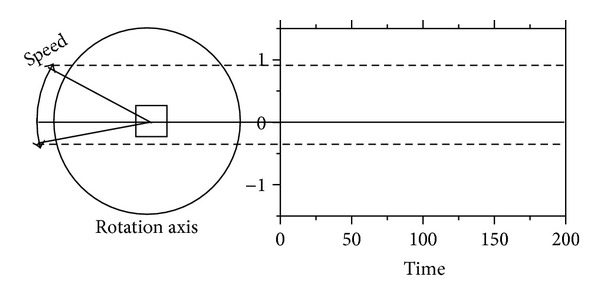
The relation between the angular velocity and the value of an oscillator.

**Figure 2:**
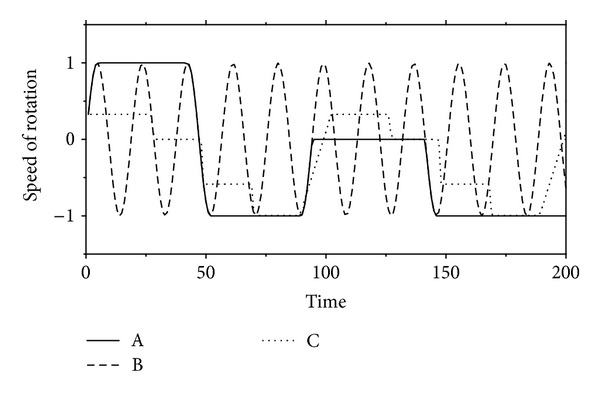
Examples of the generated patterns of an oscillator. (A) Simple rotation: the full speed to clockwise at *t*[0, 50] and to anticlockwise at *t*[50, 100]; (B) repeating the motion of a module in a sine wave; (C) a module repeating some work.

**Figure 3:**
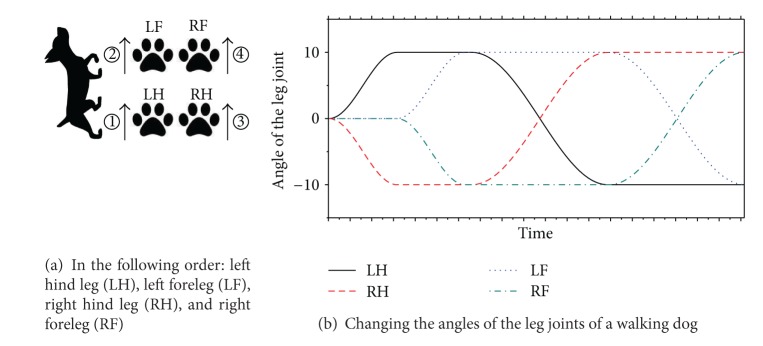
Dog walking sequence.

 As shown in Figure 1, the angular velocity of each module is defined by an absolute value of the oscillator. Usually, positive/negative values for the angles denote the rotation in clockwise/anticlockwise directions. The examples of the generated oscillations are given in Figure 2. The equation on the oscillator consists of mathematical operators: $𝔽=\{+,\;-,\;\times ,\;÷,\;{}^{\wedge },\;\sqrt{},\;\log,\;\sin(),\;\cos(),\ldots \}$. The nonlinear operators such as ${\;}^{\wedge },\;\sqrt{},\;\log,\;\sin()$, and cos() are used to represent the repetitive motion of the gaits for locomotion, as shown in Figure 2. In the simple task as given in A of Figure 2, we can easily discover a pattern apt for conducting the task. Also, the regular patterns can be made the same as the walking dog (see Figure 3). When the dog walks, he repeats the movement of his legs regularly. We can find out the pattern of the movement without making too much effort. However, it is an NP problem when considering more complex tasks or a large number of modules. In other words, the defined model (i.e., pattern) is very important, but it is difficult to find the optimal model. At this point, GP can be one of the solutions to this kind of NP problem because GP is apt for generating a complex pattern of CPG. Finally, we come up with a novel tool to generate the patterns of oscillators. We are going to explain the details of the proposed approach in Section 3.

### 2.3. Genetic Programming

**Figure 4:**
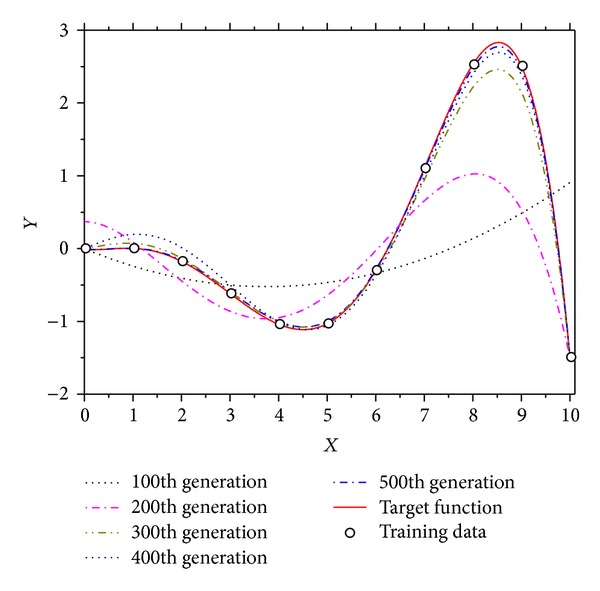
An example of symbolic regression. The error of the best individual is decreased as generation passes. The error can be calculated by summing the difference values between the actual data and the fitted data by GP.

 GP is a stochastic search mechanism inspired by the biological evolution (e.g., human's evolution) to discover computer programs by which user-defined tasks can be conducted [16]. In principle, GP individuals are a set of evolving computer programs that are represented by mathematical equation, context, grammar, and so on. Thus, GP can handle very complex, nonlinear problems such as symbolic regression. For instance, the symbolic regression problem is considered. The task is to find an optimal curve that covers accurately all the given data. As shown in Figure 4, the GP discovers a curve that accurately fits the given data as generation passes [17]. This example has shown the outstanding regression performance of the traditional GP.

In general, GP employs a nonlinear tree structure for representing individuals. The trees consist of two types of nodes: *functional* and *terminal*. The former connects the nodes below by a computer program assigned to that node and the latter is the end point consisting of input data and random values. Meanwhile, the fitness function is a measure of how well the current program has evolved. The fitness values give feedback to GP, thereby deciding which individuals are more likely to survive. After that, GP operators (i.e., selection, crossover, and mutation) are sequentially applied. Primarily, the selection narrows down the promising region in the search space. Since a tree representation is employed in GP, the crossover and the mutation are different from those of traditional evolutionary algorithms [18]. In general, GP crossover increases the exploratory power by randomly exchanging partial subtrees of parents and GP mutation maintains the search diversity by replacing a subtree of a parent with a newly generated subtree.

## 3. Proposed GP Approach to Self-Assembling Robots

 In this section, we present the proposed GP approach to the CPG for the self-assembling swarm robots. Generating the signal patterns of oscillators, which are fitted to carry out their own mission, requires very complex computation. The state of a module is influenced not only by the rotation of its adjacency modules but also by the motion of almost all modules. Existing simple solutions to these problems resort to only controlling the period and the phase of a sine wave, but they have limitations when generating more complex patterns for practical applications [4, 19]. However, the proposed GP approach is able to generate any signal pattern for each oscillator by collaboratively evolving a set of trees; thus, the self-assembling robots can precisely perform any type of action (mission). As mentioned earlier, GP has the outstanding performance in finding out a mathematical expression on complicated patterns. Thus, the multitree GP proposed herein is apt for handling the CPG problem in the self-assembling swarm robots.

### 3.1. Generating Signal Patterns

 In the self-assembling swarm robots, the most difficult but crucial task is to define their motion patterns since many motors are incorporated and the role of each motor is altered over time. The aim of this study is to discover an optimal pattern model by GP in order to control the motion direction of the module robots. To this end, the idea is to obtain the angular velocities of the modules from GP individuals. Note that each individual (of GP) is comprised of multiple trees that amount to the number of module robots. In other words, the *i*th individual consists of *N* trees in which *N* is the number of modules; *I*_*i*_ = {*T*_1_^(*i*)^, *T*_2_^(*i*)^,…, *T*_*N*_^(*i*)^}. In addition, the angular velocity of the *k*th module at the *t*th time is formulated by

(1) $$\begin{array}{c} \omega_{k,i,t}=\frac{T_{k}^{\left(i\right)}\left(x_{t}\right)}{\max\left(T\right)}\times m, \end{array}$$

where *m* is the maximum angular velocity. In this equation, the scaling term (i.e., *m*) is necessary to restrict the output value within [−*m*, *m*] since the evaluation values of trees can be too high or too low. The input data *x* consists of the information on the states of all the modules, such as the velocity information and the conjunction information.

### 3.2. Module Structure

**Figure 5:**
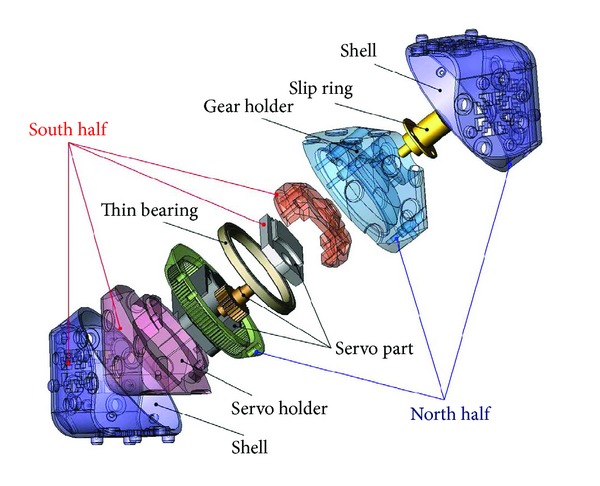
The structure of Molecube [4].

 The structure of each module is adopted from the Molecubes which are an open-source modular robotics framework [4]. As shown in Figure 5, each module robot is the same as a cube with rounded corners. It consists of two triangular pyramidal halves which are connected with their bases. Their main axes are touching each other. The halves of the cube are able to rotate around their inner motors. The module robot is equipped with an electromechanical connector at its six faces, at which other modules can be connected; thus, its degree of freedom (DOF) is three.

### 3.3. Process

 In general, the self-assembling robot system consists of a number of modules; *S* = {*M*_1_, *M*_2_,…, *M*_*N*_} where *N* is the number of modules and *M*_*i*_ = {*ω*_*i*_, *F*_*i*,1_, *F*_*i*,2_,…, *F*_*i*,6_} in which each module has one rotation axis. The angular velocity *ω*_*i*_ of the rotation axis corresponding to the *i*th module is computed from the *i*th tree of the best individual of GP.

Let *x*_*k*,*i*,*t*_ = {*ω*_*k*,1,*t*−1_, *ω*_*k*,2,*t*−1_,…, *ω*_*k*,*N*,*t*−1_, *F*_*i*,1_, *F*_*i*,2_,…, *F*_*i*,6_, *R*} be input variables for creating the *i*th tree in the *k*th individual of GP at the *t*th time. Moreover, *ω*_*k*,*i*,*t*−1_ represents the angular velocity of the *i*th module (*M*_*i*_) obtained from the *k*th individual at the (*t* − 1)th time. The binary variable of *F*_*i*,*j*_ (which is time invariant) denotes the information on whether the predefined *j*th face of *M*_*i*_ is connected with the face of any other module or not, and *R* is a random number between [0,1].

**Figure 6:**
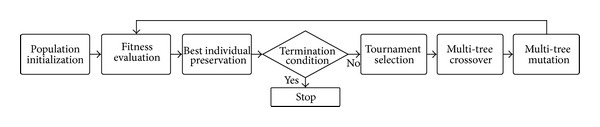
The proposed GP framework.

As shown in Figure 6, the sequence of the proposed system follows several steps: population initialization, fitness evaluation, preservation of the best individual, judgement on the termination criterion, and performing genetic operators: tournament selection, multitree crossover, and multitree mutation. The iteration of these procedures until satisfying the termination criteria makes the system evolve continually, thereby getting a powerful control model for the practical self-assembling robots. The detailed procedures are explained in the next section.

### 3.4. Representation and Evaluation

 To evaluate the individuals (i.e., trees) in GP, the functional operators in the tree are calculated along with input variables. Let *Y*_i_* ∈ *R* ^*m*^ be an optimal value with regard to the *i*th input, where *R* ^*m*^ denotes an *m*-dimensional real space. In the proposed GP, each individual consists of *N* trees where *N* is the number of modules; *I*_*i*_ = {*T*_1_^(*i*)^, *T*_2_^(*i*)^,…, *T*_*N*_^(*i*)^}. Consider a set of functions **f** = {*f*_1_, *f*_2_,…, *f*_*N*_} in which *f*_*k*_ : *T*_*k*_ → *Y*_*k*_ ∈ *R* ^*m*^. Thus, the evaluation of the *k*th tree of individuals can be performed by |*Y*_i_* − *Y*_*i*_|. Thus, the CPG problem in the self-assembling swarm robots can be formulated by

(2) $$\begin{array}{c} \arg\;\min\;f=\sqrt{\frac{1}{N}\sum_{i=1}^{N}{\left|Y_{i}^{*}-Y_{i}\right|}^{2}.} \end{array}$$

Note that the aim of GP is to discover an optimal set of functions **f** which mathematically models the signal patterns of the oscillators of all the modules in order to effectively carry out the given mission.

**Figure 7:**
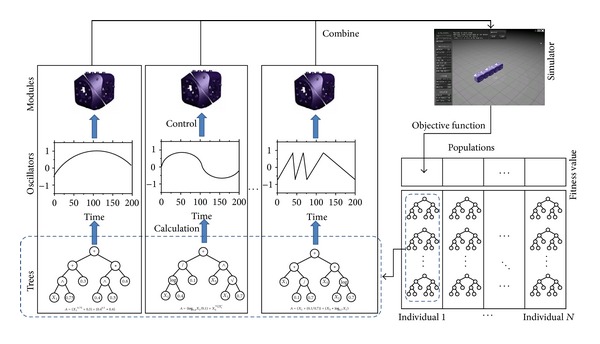
A description of applying the multitree GP to multiple modules.

As shown in Figure 7, each individual which consists of multiple trees is evaluated by computer simulations. To do this, the value obtained from the *i*th tree is first converted into the angular velocity *ω*_*i*_. And then, the velocity is utilized to control the *i*th module (*M*_*i*_) of the system. The self-assembling system controlled by these angular velocities is monitored to measure their goodness in conducting the given mission. Finally, this goodness becomes the fitness value of that individual of GP. The fitness values of all individuals are evaluated by repeating this process.

The test system (i.e., simulator) is constructed by means of the Molecubes interface [4] which employs an AGEIA PhysX physics engine and an OGRE open-source graphics engine. The goal of this study is to investigate the feasibility of the self-assembling system in various applications. Thus, the achievement factor that assesses the system performance is set by the distance of migration of the whole system. Meanwhile, the functional operators used in the proposed GP are defined as $𝔽=\{+,\;-,\;\times ,\;÷,\;{}^{\wedge },\;\sqrt{},\;\log,\;\sin(),\;\cos(),\;\text{AND},\;\text{OR},\;\text{IF-ELSE}\}$. As the terminal set, the proposed GP employs the input variables presented in Section 3.3.

### 3.5. Selection

 There are many feasible selection methods for the proposed GP, such as *τ*-wise tournament selection, roulette-wheel selection, and elitism selection. In this study, we pick up the *τ*-wise tournament selection which selects *τ* individuals in a random manner, and then, the best individual is copied into the selection pool. This selection has a higher probability of preserving the best individual which retains the highest fitness value.

### 3.6. Crossover

**Figure 8:**
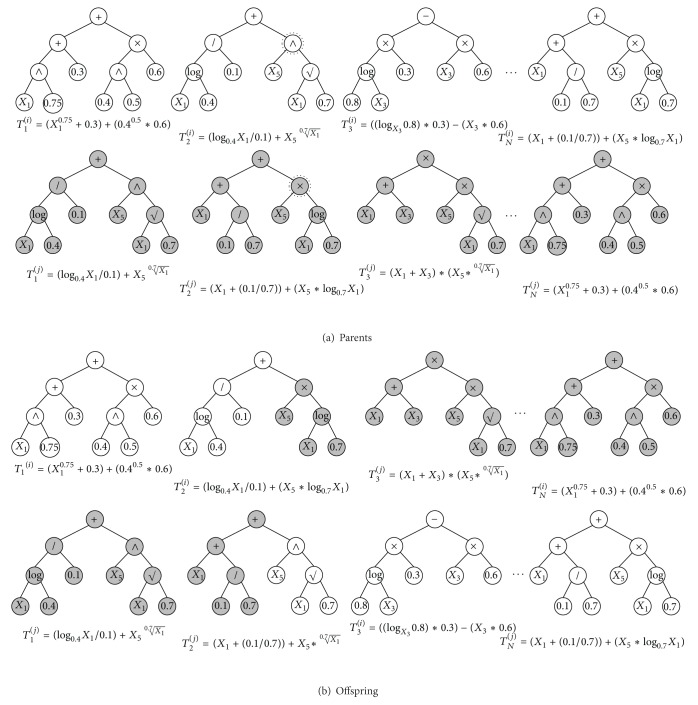
An example of the proposed GP crossover.

 Conceptually, the multitree GP crossover proposed herein exchanges the randomly selected trees or the subtrees between the parents. Although there are many alternatives to realize the crossover, we implement the proposed GP crossover similar to 1-point crossover of GA in order to preserve the well-discovered motion patterns. The GP crossover conducts two sequential mechanisms: *mixing* and *swapping*. With the two parents, more specifically, the GP crossover carries out the mixing mechanism that exchanges the subtrees of their *k*th trees at the arbitrary point and the swapping mechanism that swaps their trees from the (*k* + 1)th to the *N*th positions (see Figure 8). As usual, the subtree position for crossover is randomly chosen. For instance, consider two parent individuals as follows:

(3a) $$\begin{array}{c} I_{i}=\left\{T_{1}^{\left(i\right)},T_{2}^{\left(i\right)},\ldots ,T_{k-1}^{\left(i\right)},T_{k}^{\left(i\right)},T_{k+1}^{\left(i\right)},\ldots ,T_{N}^{\left(i\right)}\right\}, \end{array}$$

(3b) $$\begin{array}{c} I_{j}=\left\{T_{1}^{\left(j\right)},T_{2}^{\left(j\right)},\ldots ,T_{k-1}^{\left(j\right)},T_{k}^{\left(j\right)},T_{k+1}^{\left(j\right)},\ldots ,T_{N}^{\left(j\right)}\right\}. \end{array}$$

As a crossover result, the offspring *I*_*i*_′ and *I*_*j*_′ can be created as

(4a) $$\begin{array}{c} I_{i}^{\prime }=\left\{T_{1}^{\left(i\right)},T_{2}^{\left(i\right)},\ldots ,T_{k-1}^{\left(i\right)},T_{k}^{{\left(i\right)}^{\prime }},T_{k+1}^{\left(j\right)},\ldots ,T_{N}^{\left(j\right)}\right\}, \end{array}$$

(4b) $$\begin{array}{c} I_{j}^{\prime }=\left\{T_{1}^{\left(j\right)},T_{2}^{\left(j\right)},\ldots ,T_{k-1}^{\left(j\right)},T_{k}^{{\left(j\right)}^{\prime }},T_{k+1}^{\left(i\right)},\ldots ,T_{N}^{\left(i\right)}\right\}, \end{array}$$

where *T*_*k*_^(*i*)′^ and *T*_*k*_^(*j*)′^ represent the *k*th trees of the *i*th and the *j*th offspring after the mixing mechanism, respectively.

### 3.7. Mutation

**Figure 9:**
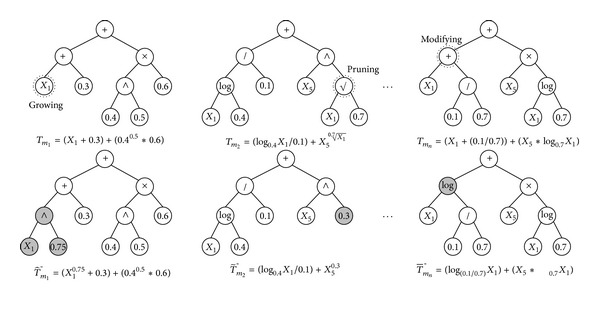
An example of the proposed GP mutation.

 In principle, mutation randomly alters some nodes of a chosen tree. As shown in Figure 9, the proposed GP mutation consists of three mechanisms: *pruning*, *growing*, and *modifying*. In the pruning case, a subtree from an arbitrary functional node is replaced with an arbitrary leaf (i.e., terminal) node. In the growing case, an arbitrary leaf node is replaced with a randomly created subtree. In the modifying case, an arbitrary functional node is changed by another one. For instance, an *i*th individual is given as

(5) $$\begin{array}{c} I_{i}=\left\{T_{1},T_{2},\ldots ,T_{m_{1}},\ldots ,T_{m_{2}},\ldots ,T_{m_{n}},\ldots ,T_{N-1},T_{N}\right\}. \end{array}$$

The mutation produces an offspring as follows:

(6) $$\begin{array}{c} I_{i}^{\prime \prime }=\left\{T_{1},T_{2},\ldots ,{\hat{T}}_{m_{1}},\ldots ,{\tilde{T}}_{m_{2}},\ldots ,{\overset{-}{T}}_{m_{n}},\ldots ,T_{N-1},T_{N}\right\}, \end{array}$$

where *m*_1_, *m*_2_,…, *m*_*n*_ are randomly chosen and $\hat{T}$, $\tilde{T}$, and $\overset{-}{T}$ denote the results of the growing, the pruning, and the modifying mechanisms, respectively.

In the mutation process, one of these adding nodes (i.e., growing), removing nodes (i.e., pruning), and changing nodes (i.e., modifying) is settled, and then, a mutation point is chosen in a random fashion. The decided mechanism is performed at the mutation point. For instance, if the growing mechanism is conducted at a leaf node, the node type of the selected position is changed from “terminal” to “functional,” and then, a randomly generated subtree is inserted into that position.

## 4. Experimental Results

**Figure 10:**
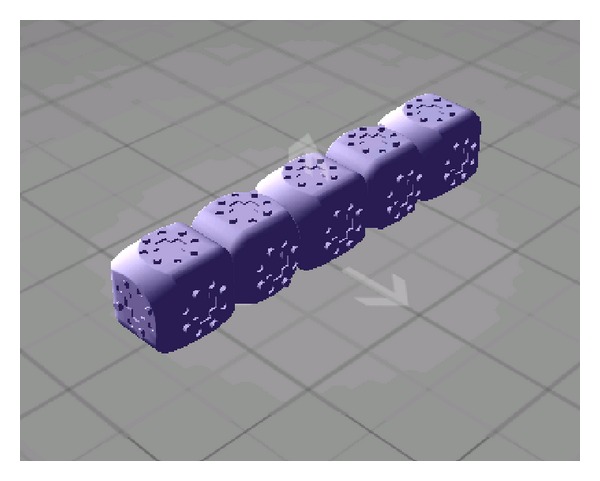
The initial configuration of the system.

 The proposed approach was tested by a computational experiment in the physical environment. As mentioned earlier, the simulator was constructed on the Molecubes interface using the PhysX and OGRE engines. The goal was to move the robot system as far as possible on the ground within 200 seconds. Only focusing on the effectiveness of the proposed approach, a smaller number of robot modules were employed in this experiment. As shown in Figure 10, moreover, the initial configuration of the system was fixed by the combined five modules which are sequentially sitting on the flat ground. Assume that the gravity is the same as the earth's one and the air resistance is ignored. If a module cannot rotate on the axis due to the impediments, the module stops its rotation. Moreover, the breakdown of the modules was not considered in this experiment.

For the parameter setting, the population size is 200, the maximum number of generations is 200, and the pairwise tournament selection is used. Moreover, the probabilities of crossover and mutation (i.e., *P*_*c*_ and *P*_*m*_) are set to 0.8 and 0.2, respectively. These values were determined by the empirical analysis. In addition, the elitism was used to preserve the best individual discovered so far. The initial individuals were randomly generated under the depth limit of ten nodes; each tree was comprised of approximately two hundred nodes. In this experiment, five modules were deployed (i.e., *N* = 5). The functional set was defined as $𝔽=\{+,\;-,\;\times ,\;÷,\;{}^{\wedge },\;\sqrt{},\;\log\}$ and the terminal set was given as *𝕋* = {*ω*_1,*t*_, *ω*_2,*t*_,…, *ω*_5,*t*_, *F*_*i*,1_, *F*_*i*,2_,…, *F*_*i*,6_, *R*} (see Section 3.3). In the case of “÷,” the protected division was used to avoid the error when a numerator is divided by zero. When *t* is “zero” (i.e., the initial state), the rotation axes of all modules are at a stop; thus, we set *ω*_*k*,*t*=0_ = 0 and *ω*_*k*,*t*=−1_ = 0, for  all  *k*.

**Figure 11:**
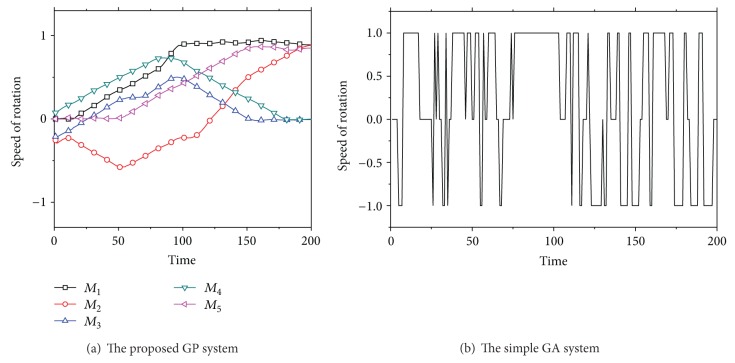
The generated signal patterns of the oscillators by the best individual at the 200th generation.

**Figure 12:**
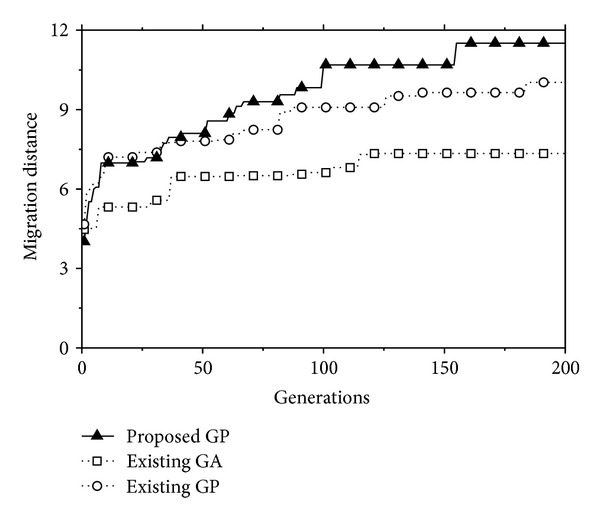
The migration distance achieved by the best individual for 200 seconds as generation progresses.

In Figure 11(a), the signal patterns generated on the five modules (i.e., *M*_1_, *M*_2_, *M*_3_, *M*_4_, and *M*_5_) were obtained by the equation of each tree in the best individual at the 200th generation. Then, the self-assembling system utilized the obtained patterns. As shown in Figure 12, the proposed system could move ahead about 12 meters from the starting position during 200 seconds. In this figure, we could also find that the proposed system is evolving because its migration distance continuously increases from approximately 4 meters at the first generation to about 12 meters at the 200th generation. Note that the results in Figure 12 were averaged over ten runs in order to take into account the stochastic nature of GP.

To assure the effectiveness of the proposed GP approach, we performed a comparative experiment in which a simple GA [18] was employed. In the GA system, each module had three states of the angular velocity: {−*m*, 0, *m*}. An individual was comprised of five chromosomes since five modules were used in this experiment. Each chromosome in the GA individual was set to 200 units, which represents the angular velocity of a module at every second. The parameter setting of GA was given as follows: the population size and the number of generations were set to 200 and 200, respectively, the pair-wise tournament selection was used, and 1-point crossover and uniform mutation were applied with their probabilities of 0.7 and 0.05, respectively. They were naturally decided by the empirical observation. The elitism was employed as well. The generated signal from the best individual in the GA system is shown in Figure 11(b). In this figure, we only plotted one signal pattern among the generated signals due to the difficulties in visualizing many overlapping signals. It was also observed that the GA system gradually improves their performance since the migration distance achieved by the GA system changes from around 4 meters at the first generation to about 7 meters at the last generation (see Figure 12). Nonetheless, this result showed that the performance of the proposed GP approach is much better than the that of GA system at all generations. While the GA system got stuck completely after the 120th generation, the proposed GP system continuously improved its performance. This implies that the proposed GP approach becomes more and more efficient as generation passes, as compared with the GA system.

For the purpose of comparison, another experiment was conducted on the existing GP approach [19]. The parameter setting used in the proposed GP approach was employed in the existing GP system. In Figure 12, it was shown that the migration distance of the existing GP system amounts to around 10 meters at the end of the generation: the proposed GP system moves faster and further than the existing GP system.

**Figure 13:**
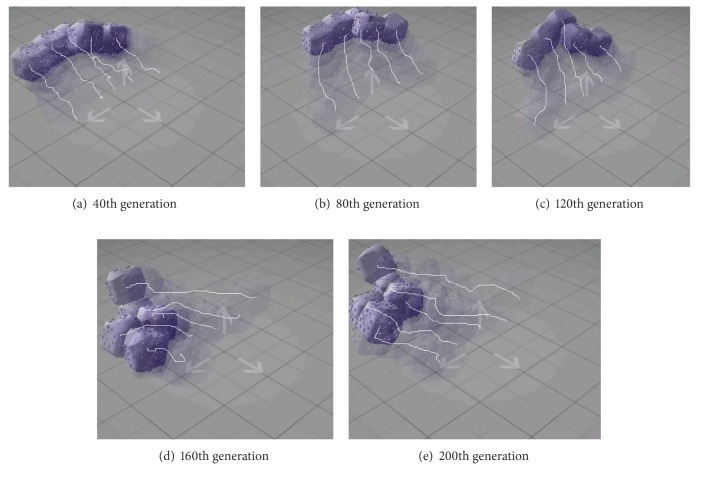
The movement traces of the proposed system as generation passes.

On the other hand, the movement traces in Figure 13 demonstrate that the structure of the proposed system was transformed into a suitable shape to carry out their task. For instance, the system in Figure 13(a) tried to move as far as possible by means of a structure similar to the initial one. As time goes on, the shape of the proposed system was able to be transformed into a more complex structure suitable to move faster and further than the earlier systems.

## 5. Conclusion

 In this paper, we proposed a control algorithm for the self-assembling swarm robot system. The main idea was to generate the signal patterns of oscillators by means of GP in order to perform the locomotion of the system. To this end, new multitree GP that is apt for generating the signal patterns of robot modules for the locomotion was developed. The experimental results showed that the proposed system achieves acceptable performance due to its evolutionary nature. Moreover, the proposed GP approach outperformed the existing GA and GP methods. Although there were limitations in the experiment, such as the time-consuming simulation, a few modules, a smaller population size, and the small number of generations, the proposed system sufficiently showed great promise to design the oscillators promising for the locomotion of the self-assembling robot system.

As the future work, we are going to improve the processing speed of the proposed system to overcome the aforementioned limitations. In terms of scalability, the system can be enhanced by incorporating the domain-specific knowledge into the crossover and mutation operators. In addition, we will make progress on the research on other types of task, such as jumping, swimming, and running. It is expected that the proposed GP-based self-assembling swarm robot system provides a new gait of locomotion in carrying out their mission.
